# Hollow nanoparticles synthesized via Ostwald ripening and their upconversion luminescence-mediated Boltzmann thermometry over a wide temperature range

**DOI:** 10.1038/s41377-022-00867-9

**Published:** 2022-07-11

**Authors:** Ran An, Yuan Liang, Ruiping Deng, Pengpeng Lei, Hongjie Zhang

**Affiliations:** 1 State Key Laboratory of Rare Earth Resource Utilization, Changchun Institute of Applied Chemistry, Chinese Academy of Sciences, 5625 Renmin Street, 130022 Changchun, China; 2 University of Science and Technology of China, 230026 Hefei, China; 3 Ganjiang Innovation Academy, Chinese Academy of Sciences, 341000 Ganzhou, Jiangxi China; 4 Department of Chemistry, Tsinghua University, 100084 Beijing, China

**Keywords:** Nanoparticles, Optical sensors

## Abstract

Upconversion nanoparticles (UCNPs) with hollow structures exhibit many fascinating optical properties due to their special morphology. However, there are few reports on the exploration of hollow UCNPs and their optical applications, mainly because of the difficulty in constructing hollow structures by conventional methods. Here, we report a one-step template-free method to synthesize NaBiF_4_:Yb,Er (NBFYE) hollow UCNPs *via* Ostwald ripening under solvothermal conditions. Moreover, we also elucidate the possible formation mechanism of hollow nanoparticles (HNPs) by studying the growth process of nanoparticles in detail. By changing the contents of polyacrylic acid and H_2_O in the reaction system, the central cavity size of NBFYE nanoparticles can be adjusted. Benefiting from the structural characteristics of large internal surface area and high surface permeability, NBFYE HNPs exhibit excellent luminescence properties under 980 nm near-infrared irradiation. Importantly, NBFYE hollow UCNPs can act as self-referenced ratiometric luminescent thermometers under 980 nm laser irradiation, which are effective over a wide temperature range from 223 K to 548 K and have a maximum sensitivity value of 0.0065 K^−1^ at 514 K. Our work clearly demonstrates a novel method for synthesizing HNPs and develops their applications, which provides a new idea for constructing hollow structure UCNPs and will also encourage researchers to further explore the optical applications of hollow UCNPs.

## Abstract (web summary)

Hollow UCNPs with tunable cavity size were synthesized, which can be used as ratiometric luminescent thermometers over a wide temperature range.

## Graphical abstract

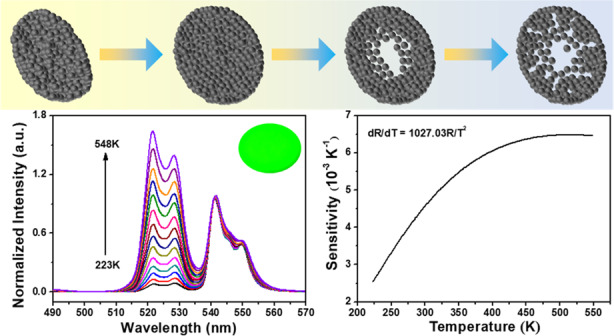

## Introduction

As one of the most common physical parameters in scientific and industrial applications, the accurate measurement of temperature has always been a concern for researchers^1^. Luminescence thermometry is a method of measuring temperature by using luminescence properties that are highly dependent on temperature changes, such as luminescence intensity or fluorescence lifetime^2^. It shows broad application prospects because of the advantages of high sensitivity, fast response, non-contact, large spatial resolution, and tolerance to extreme conditions^3–5^. In particular, ratiometric luminescent thermometers that rely on changes in intensity ratios between emission bands to measure temperature are self-referenced and simple because they do not require additional calibration of emission intensity^6^. Such a thermometer is independent of the inhomogeneity and concentration of the luminescent centers in the materials. However, the application of ratiometric luminescent thermometers with excitation wavelengths in the ultraviolet-visible region is limited, especially in the biological field. Moreover, the stability of materials seriously affects the temperature measurement range of luminescent thermometers.

Ratiometric luminescent thermometers based on upconversion luminescence (UCL) have received widespread interest because of their high sensitivity and low-energy near-infrared (NIR) photoexcitation properties^7,8^. Among the numerous UCL materials, lanthanide-doped upconversion nanoparticles (UCNPs) stand out and become gradually one of the most common advanced functional nanomaterials due to their excellent optical, magnetic and physicochemical properties^9–13^. In the past decades, researchers have developed a variety of lanthanide-doped UCNPs and confirmed that this type of materials have many superior properties, including long lifetimes, tunable colors, superior photostability, large anti-Stokes shifts, and weak autofluorescence^14–16^. These outstanding advantages ensure the application of UCNPs for temperature sensing over a wide temperature range^17–19^. In addition, UCNPs have also been used as a platform to construct composite functional materials^20–24^, further broadening their applications in temperature sensing. Among common lanthanide UCL ions, Er^3+^ ions, of which two emission bands produced by the transitions of ^2^H_11/2_ → ^4^I_15/2_ and ^4^S_3/2_ → ^4^I_15/2_ have high sensitivity to temperature changes, have undoubtedly received the most attention in the previous studies^25^. The redistribution of energy between the excited states ^2^H_11/2_ and ^4^S_3/2_ of Er^3+^ ions obey the Boltzmann-type distribution. Importantly, the ratios of the luminescence intensities attributed to radiation transitions from the above-mentioned two excited states back to ^4^I_15/2_ are not affected by fluorescence loss and excitation intensity fluctuations, thereby minimizing the influence of external conditions on the temperature measurement process^26,27^. These merits make ratiometric luminescent thermometers based on UCNPs very promising in the field of temperature sensing.

Recently, various shapes of UCNPs, such as spheres, cubes, cylinders, nanowires, dumbbells, plates, etc., have been meticulously synthesized^28–30^, but there are few studies on hollow nanoparticles (HNPs). Hollow inorganic nanoparticles featuring a cavity space are one of the most attractive functional nanomaterials^31,32^. Hollow structure UCNPs have the properties of large internal surface area and high surface permeability, and theoretically have high light collection efficiency that is conducive to obtaining excellent luminescence properties and thus beneficial to temperature sensing^33,34^. However, the controllable design, structural engineering, and fine-tuning of nanoparticles to construct special structural materials with desirable size, composition, morphology, and physicochemical properties are not an easy task^35–37^. Common synthesis strategies for preparing hollow structures mainly include the hard template method, microfluidic method, soft template method, and spraying method^38–41^. In particular, the synthesis of hollow materials by the hard template method has received lots of attention from researchers. It usually requires post-etching processing, which is not only time consuming, but also undoubtedly increases the cumbersomeness of the experiment^42^. Especially for optical materials whose luminescence performance is closely related to defects^43–45^, the post-processing process may reduce the stability of the materials and increase the defects, which will have a significant impact on the luminescence of materials. Recently, the template-free one-step method through Ostwald ripening mechanism has shown great advantages and application prospects in fabrication of HNPs due to its process simplicity^46^.

In this study, we have synthesized NaBiF_4_:Yb,Er (NBFYE) hollow UCNPs by Ostwald ripening under a template-free one-step solvothermal method. Furthermore, the possible mechanism of the formation of HNPs was carefully analyzed. Under controllable synthesis conditions, the size of the cavity in the nanoparticles can be precisely adjusted. The luminescence performance of NBFYE HNPs was studied under NIR laser excitation. In addition, the ratiometric luminescent temperature sensing properties of NBFYE hollow UCNPs were systematically investigated in the temperature range of 223–548 K. The work presents a novel method to prepare HNPs and further explores the optical application of hollow UCNPs in the field of luminescent temperature sensing.

## Results

### Morphology, structure, and composition

**Fig. 1:**
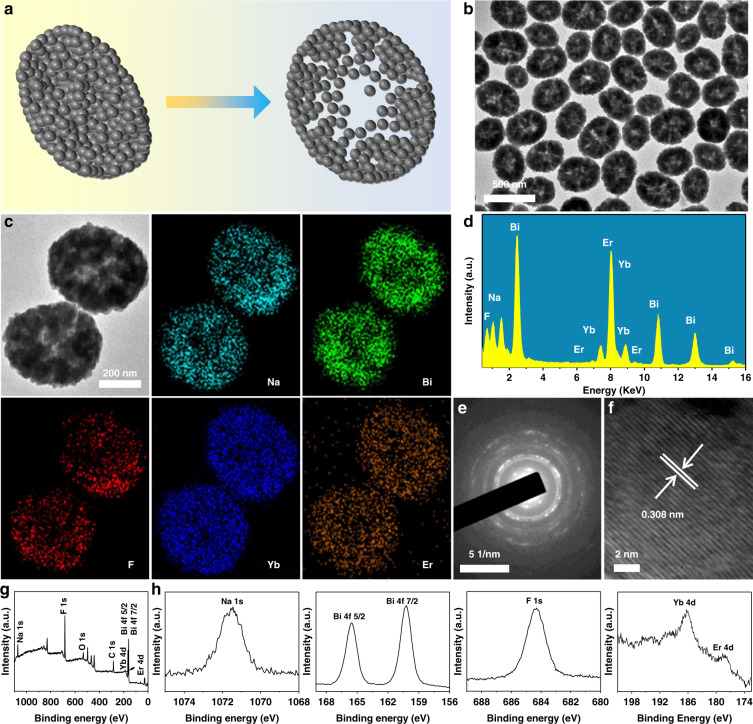
Structural characterization of NBFYE HNPs synthesized by adding 1.5 g PAA and 5 mL H_2_O to the reaction system. **a** Schematic diagram of the structural change from solid to hollow. **b** TEM image. **c** Elemental mapping images. **d** EDS spectra. **e** SAED pattern. **f** HRTEM image. XPS spectra: (**g**) survey spectrum, and (**h**) high-resolution spectra of Na 1s, Bi 4f, F 1s, Yb 4d, and Er 4d

The transformation of NBFYE nanoparticles from solid to hollow is illustrated in Fig. 1a. As indicated, NBFYE nanoparticles are comprised of many smaller crystallites. Figure 1b displays the transmission electron microscopy (TEM) image of NBFYE HNPs. Furthermore, the elemental mapping images of two randomly selected nanoparticles verify that all existing elements are mainly distributed at the edge of the nanoparticles, confirming the formation of the hollow structure and uniform doping of lanthanide elements Yb and Er in NaBiF_4_ (Fig. 1c). Moreover, the existence of the five elements in NBFYE HNPs was also proved by energy-dispersive X-ray spectroscopy (EDS) spectra (Fig. 1d). As shown in Fig. 1e, f, the selected area electron diffraction (SAED) pattern and high-resolution TEM (HRTEM) image of HNPs confirm its good crystalline property with hexagonal structure. In addition, the chemical composition of NBFYE HNPs was analyzed by X-ray photoelectron spectroscopy (XPS), and the high-resolution spectra of these elements were measured to determine their oxidation states (Fig. 1g, h). There are six distinct peaks in the spectrum from high to low binding energy, which correspond to Na 1 s (1071.5 eV), F 1 s (684.3 eV), Yb 4d (186.3 eV), Er 4d (178.6 eV), Bi 4f_5/2_ (165.5 eV), and Bi 4f_7/2_ (160.2 eV), respectively. The oxidation states of elements Yb, Er, and Bi are all +3. All the above results confirmed the successful preparation of NBFYE HNPs.

### Growth process and formation mechanism

**Fig. 2:**
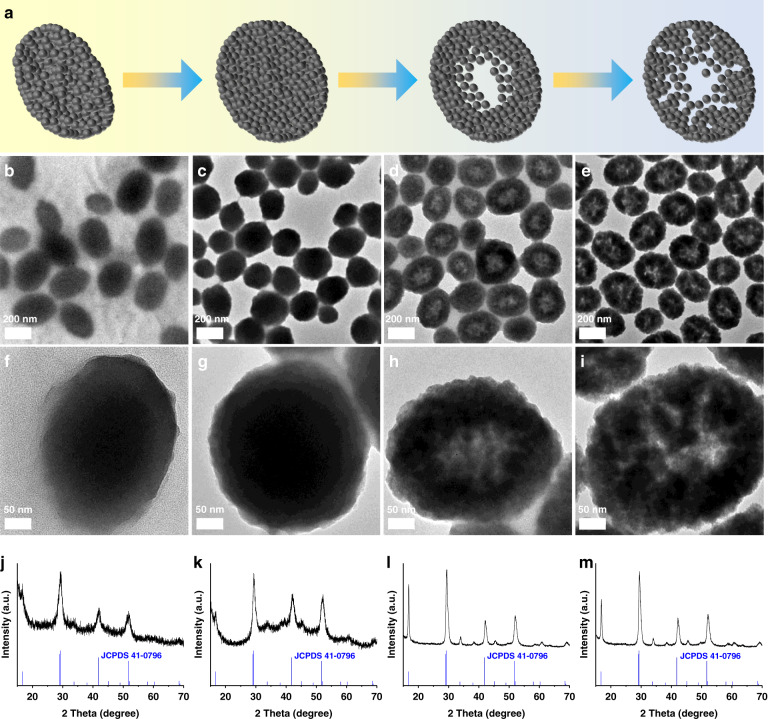
Structural characterization of NBFYE nanoparticles synthesized at different reaction times. **a** Schematic illustration of the morphological evolution of nanoparticles over time. TEM images (**b**–**i**) and XRD patterns (**j**–**m**) of NBFYE nanoparticles synthesized by adding 1.5 g PAA and 5 mL H_2_O to the reaction system for different reaction times. (**b**, **f**, **j**) 0 h, (**c**, **g**, **k**) 1 h, (**d**, **h**, **l**) 5 h, and (**e**, **i**, **m**) 9 h

The formation mechanism of NBFYE HNPs was investigated. Figure 2a shows the evolution of the morphology of nanoparticles over time. In the first hour at the beginning of the reaction, the obtained NBFYE nanoparticles are solid (Fig. 2b, c). As the reaction time increases, cavities appear in the middle of the nanoparticles, and the hollowing becomes the major process. As shown in Fig. 2d, e, NBFYE nanoparticles with hollow structure can be clearly observed. Moreover, NBFYE nanoparticles change from solid to hollow and the inner space becomes increasingly larger, which can be further observed from TEM image of a single nanoparticle (Fig. 2f–i). In addition, X-ray diffraction (XRD) results of nanoparticles synthesized at different times could all be indexed to the hexagonal-phase NaBiF_4_ (Fig. 2j–m), and it is also confirmed that the crystallinity of nanoparticles becomes better and better with the prolongation of reaction time. Compared with standard hexagonal-phase NaBiF_4_, the XRD peaks of NBFYE move slightly to high-angle side because the Yb^3+^/Er^3+^ ions with smaller radius replace the Bi^3+^ ions (*r* = 1.31 Å) with larger radius (Fig. S1).

Based on the above observations, it can be inferred that the production of central cavity is mainly mediated by the Ostwald ripening process. At reaction times of 5 to 9 h, the hollow interiors become larger, and the size of the nanoparticles increases slightly. This is due to the occurrence of the Ostwald ripening process, that is, the high-energy nanocrystals located in the central part dissolve and re-deposited on the outside of the nanoparticles. During the reaction time of 9 to 29 h, the HNPs still maintained their morphology, but their size gradually increased (Figs. 2e and S2a–c), indicating that the Ostwald ripening process continued during this period. With the further prolongation of the reaction time to 97 h, the morphology of NBFYE nanoparticles changed significantly, and the hollow structure was destroyed and disintegrated (Fig. S2d). However, the nanoparticles always maintain the pure hexagonal-phase structure, and no impurity peaks appear (Fig. S2e–h).

On the basis of the above analysis and explanation, we conducted relevant comparative experiments to further carefully study the growth process of NBFYE HNPs. In the absence of polyacrylic acid (PAA), solid nanoparticles were obtained regardless of whether H_2_O was introduced into the reaction solution (Fig. S3). Therefore, it is reasonable to speculate that PAA is essential and indispensable for the formation of HNPs. Then, the amount of H_2_O added to the reaction solution was fixed at 5 mL, and we changed the amount of PAA to study the morphological changes of nanoparticles. As shown in Fig. S4, the inner space of the obtained nanoparticles is very small when the amount of PAA is 0.5 g. As the amount of PAA increases, the inner space of the obtained nanoparticles increases, and there is no obvious difference between the HNPs obtained with the amount of PAA of 1.5 g and 2.0 g. Moreover, Fourier transform infrared spectroscopy (FT-IR) reveals absorption peaks attributed to PAA molecules, indicating that the presence of PAA in the resulting NBFYE HNPs (Fig. S5). The above results verified that PAA is crucial for the formation of NBFYE HNPs.

**Fig. 3:**
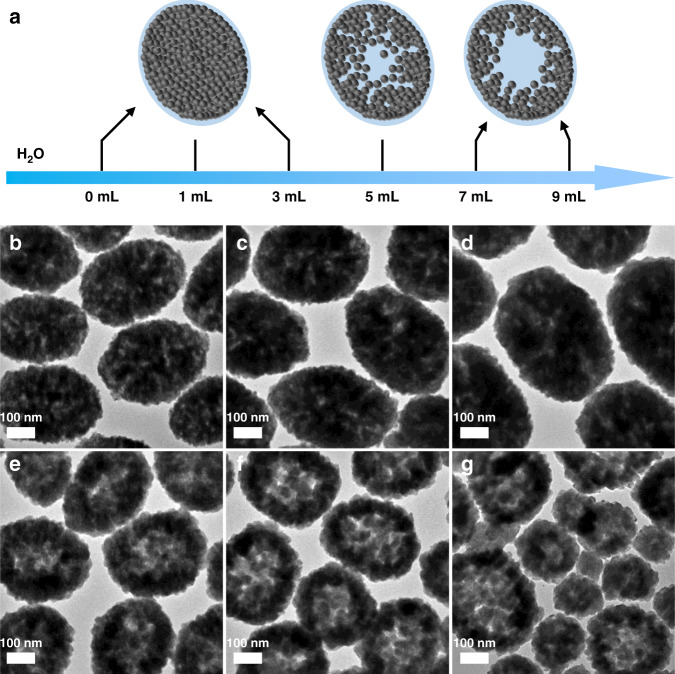
Structural characterization of NBFYE nanoparticles synthesized by adding different amounts of H_2_O to the reaction system. **a** Schematic illustration of the morphology of nanoparticles varying with the amount of H_2_O. TEM images of NBFYE nanoparticles synthesized by adding different amounts of H_2_O to the reaction system in the presence of 1.5 g PAA. (**b**) 0 mL, (**c**) 1 mL, (**d**) 3 mL, (**e**) 5 mL, (**f**) 7 mL, and (**g**) 9 mL

Then, we investigated the role of H_2_O in the construction of HNPs in the presence of 1.5 g PAA in the reaction system. The entire evolution process of the nanoparticle morphology with the amount of H_2_O added is demonstrated in Fig. 3a. When the amount of H_2_O varies from 0 to 3 mL, no obvious hollow interior appears (Fig. 3b–d). As the amount of H_2_O increased to 5 mL, the hollow structure of the nanoparticles appeared obviously (Fig. 3e). After further increasing the amount of H_2_O to 7 mL, the inner space of the nanoparticles is further increased compared to the edges (Fig. 3f). The inner space of the nanoparticles obtained by adding 9 mL H_2_O to the solution did not change significantly compared with the nanoparticles obtained by adding 7 mL H_2_O, but the morphology became slightly irregular (Fig. 3g). In addition, the XRD results showed that all samples had no impurity peaks and maintained the hexagonal structure of NaBiF_4_ (Fig. S6). To sum up, it can be confirmed that the simultaneous presence of PAA and H_2_O is necessary for the formation of HNPs, and their amounts have obvious effects on the internal cavity size of NBFYE HNPs.

### Luminescence properties

**Fig. 4:**
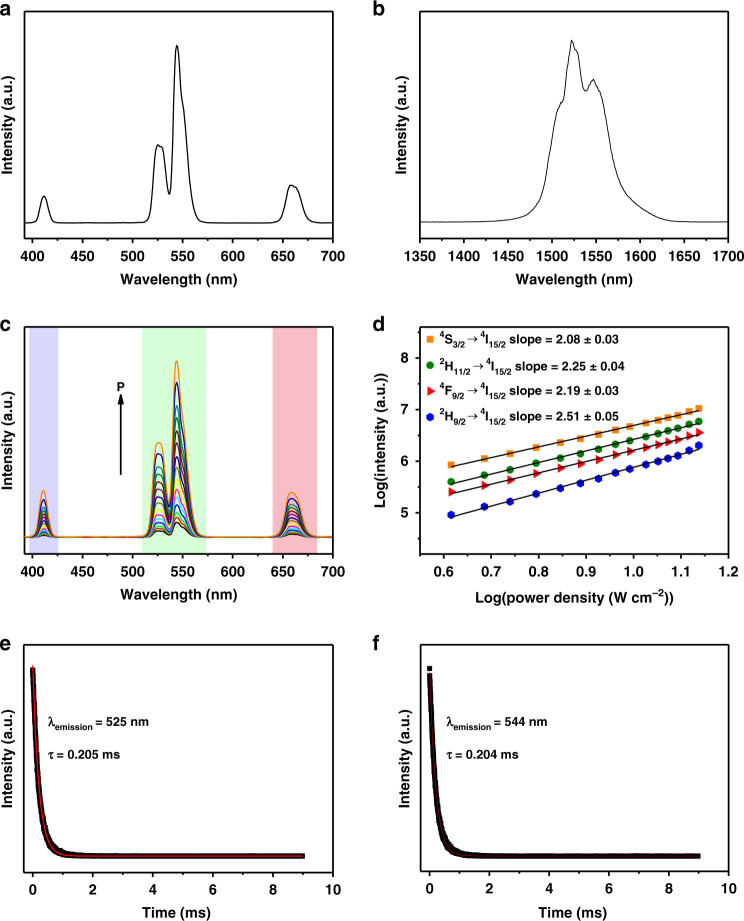
Fluorescence spectra of NBFYE HNPs synthesized by adding 1.5 g PAA and 5 mL H_2_O to the reaction system under 980 nm irradiation. **a** UCL and (**b**) NIR-II spectra (*P* = 4.85 W cm^−2^). **c** UCL spectra with excitation laser power between 4.13 W cm^−2^ and 13.73 W cm^−2^. **d** Pump power dependence of UCL intensity. Time-resolved fluorescence decays of Er^3+^ ions emission at (**e**) 525 nm and (**f**) 544 nm

The upconversion/downshifting fluorescence spectra of NBFYE HNPs synthesized by adding 1.5 g PAA and 5 mL H_2_O to the reaction system were recorded under 980 nm NIR irradiation. Figure 4a, b display the abundant characteristic peaks of Er^3+^ ions from blue light emission (411 nm, ^2^H_9/2_ → ^4^I_15/2_), green (525 nm, ^2^H_11/2_ → ^4^I_15/2_; 544 nm, ^4^S_3/2_ → ^4^I_15/2_), red (658 nm, ^4^F_9/2_ → ^4^I_15/2_), to NIR (1522 nm, ^4^I_13/2_ → ^4^I_15/2_). Then, the variation of UCL of NBFYE HNPs with laser power density (*P*) was systematically investigated. Figure 4c shows that the UCL intensity (*I*) increases as *P* increases. For upconversion, when the n-photon pumping process occurs, there is an nth power function relationship between *I* and *P*, that is, *I* ∝ *P*^n^^47,48^. From Fig. 4d, it can be observed that the fitted slopes of n for ^2^H_9/2_ → ^4^I_15/2_, ^2^H_11/2_ → ^4^I_15/2_, ^4^S_3/2_ → ^4^I_15/2_, and ^4^F_9/2_ → ^4^I_15/2_ are 2.51 ± 0.05, 2.25 ± 0.04, 2.08 ± 0.03, and 2.19 ± 0.03, respectively. In addition, the decay curves of UCL of Er^3+^ ions are demonstrated in Figs. 4e, f, and S7. All of them can be fitted by the single exponential function: *I*(t) = *I*_0_ + *A*_1_exp(−*t/τ*_1_), where *τ*_1_ is the fluorescence lifetime, *I*(t) and *I*_0_ represent the fluorescence intensities at time *t* and 0, and *A*_1_ is a constant^49^. The average lifetimes of NBFYE HNPs at 411 nm, 525 nm, 544 nm, and 658 nm are 0.108 ms, 0.205 ms, 0.204 ms, and 0.317 ms, respectively. Among them, the emission at 525 nm and 544 nm have almost the same fluorescence lifetimes, which is expected for the thermally coupled excited states^50^.

When Yb/Tm or Yb/Ho is doped with NaBiF_4_, the formed NaBiF_4_:Yb,Tm and NaBiF_4_:Yb,Ho nanoparticles exhibit their own characteristic UCL (Figs. S8 and 9). Then, the variation of their emission spectra with *P* was also studied. For NaBiF_4_:Yb,Tm UCNPs, the fitted slope value is 1.96 ± 0.12, indicating that the pump photon number for NIR emission is 2 (Fig. S10). The n values for the ^5^F_5_ → ^5^I_8_ and ^5^F_4_,^5^S_2_ → ^5^I_8_ of NaBiF_4_:Yb,Ho UCNPs are 1.97 ± 0.04 and 1.85 ± 0.03, respectively (Fig. S11). Moreover, the fluorescence lifetimes of the two luminescent nanoparticles were also tested. As displayed in Figs. S12 and 13, the average decay time of NaBiF_4_:Yb,Tm at 808 nm is 0.384 ms, and the fluorescence lifetimes of NaBiF_4_:Yb,Ho at 540 nm and 646 nm are 0.282 ms and 0.283 ms, respectively.

### Temperature-dependent luminescence properties

**Fig. 5:**
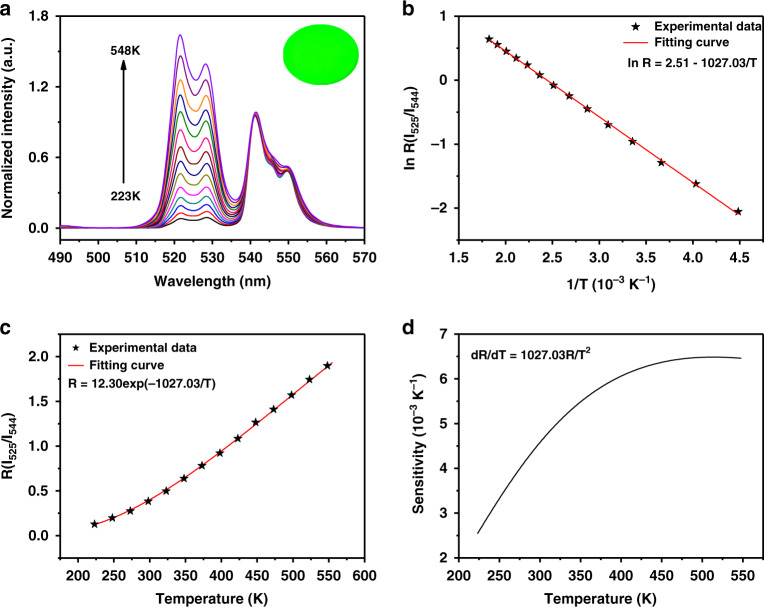
Temperature-dependent luminescence properties of NBFYE HNPs synthesized by adding 1.5 g PAA and 5 mL H_2_O to the reaction system. **a** Normalized emission spectra of NBFYE HNPs as a function of temperature upon NIR irradiation (*P* = 4.85 W cm^−2^). The inset in the upper right corner shows the luminescence photograph of HNPs under 980 nm NIR irradiation at room temperature. **b** The linear relationship between the natural logarithm of *R* (*I*_525_/*I*_544_) vs 1/T. **c** *R* (*I*_525_/*I*_544_) as a function of temperature. **d** The sensing sensitivity of NBFYE HNPs varies with temperature from 223 K to 548 K

The relative fluorescence intensities of the two green-emitting radiative transitions of Er^3+^ ions have a strong temperature dependence, which is attributed to the thermally coupled ^2^H_11/2_ and ^4^S_3/2_ states. Under NIR laser irradiation, the ratiometric luminescent temperature sensing performance of NBFYE HNPs synthesized by adding 1.5 g PAA and 5 mL H_2_O to the reaction system was systematically investigated. As displayed in Fig. 5a, normalized to the maximum peak intensity at 544 nm, the fluorescence peak at 525 nm keeps rising with increasing temperature. The inset in the upper right corner of Fig. 5a shows the corresponding luminescence photograph of HNPs under 980 nm NIR irradiation, confirming their excellent luminescence. With the change of temperature, the ratio (*R*) of the luminescence intensity of two emissions centered at 525 nm (*I*_525_) and 544 nm (*I*_544_) can be defined as described by Eq. (1).

1 $$R = \frac{{I_{525}}}{{I_{544}}} = C{{{\mathrm{exp}}}}\left( { - \frac{{\Delta E}}{{kT}}} \right)$$

where *T* is the Kelvin temperature, Δ*E* is the energy gap between the ^2^H_11/2_ and ^4^S_3/2_ levels, *C* is the pre-exponential factor, and *k* is the Boltzmann constant.

Figure 5b presents a good linear relationship between ln *R* and 1/*T*. The slope value (Δ*E/k*) is 1027.03, so the calculated Δ*E* is 711.5 cm^−1^, which is approximately the energy gap between the ^2^H_11/2_ and ^4^S_3/2_. As shown in Fig. 5c, *R* exhibited a good exponential relationship with temperature between 223 K and 548 K. Subsequently, we studied the sensitivity of NBFYE HNPs as sensors in detail. Eq. (2) can be used to calculate the sensitivity.

2 $$S = \frac{{{\rm{d}}R}}{{{\rm{d}}T}} = R\left( {\frac{{\Delta E}}{{kT^2}}} \right)$$

As can be seen from Fig. 5d, the sensitivity first increases and then remains basically unchanged with the increase of temperature, and there is no significant difference between 490 and 548 K, suggesting that NBFYE HNPs are more supportive for temperature measurements in high-temperature range. The sensitivity reaches a maximum of 0.0065 K^−1^ at 514 K. Importantly, NBFYE HNPs can maintain the hexagonal-phase structure even after heating at a high temperature of 548 K (Fig. S14), confirming that they are very stable. The results verified that NBFYE HNPs could be used as stable ratiometric luminescent thermometers over a wide temperature range.

## Discussion

In conclusion, we have successfully prepared NBFYE hollow UCNPs under a template-free one-step solvothermal method. Ostwald ripening process is the main mechanism for the formation of HNPs. With the adjustment of the amount of PAA and H_2_O, the central cavity size of HNPs can be controlled. Under 980 nm laser irradiation, NBFYE HNPs show excellent luminescence properties due to the inherent prominent advantages of hollow structure including large internal surface area and high surface permeability. The ratiometric luminescent temperature sensing performance of NBFYE hollow UCNPs in the range of 223–548 K demonstrates that they can be used as self-referenced luminescent thermometers over a wide temperature range, with a maximum sensitivity of 0.0065 K^−1^ (514 K). Our work proposes a facile and useful strategy to synthesize hollow UCNPs, and also clearly demonstrates the formation mechanism of HNPs, and the obtained NBFYE HNPs could be applied for ratiometric luminescent temperature sensing.

## Materials and methods

### Chemicals and materials

Ethylene glycol (EG, ≥ 99%), PAA (M.W. 1800 g mol^−1^), NaNO_3_ (99.0%), Bi(NO_3_)_3_·5H_2_O, (98.0%), Ln(NO_3_)_3_·5H_2_O (99.9%, Ln = Er, Ho, Tm, and Yb), and NH_4_F (99.99%) were purchased from Sigma-Aldrich. All reagents were used as received without further purification.

### Preparation of NBFYE HNPs

A typical preparation process of NBFYE HNPs is described as follows. First, lanthanide stock solution A (aqueous solution) with concentrations of 18.0 g L^−1^ Yb(NO_3_)_3_·5H_2_O and 1.8 g L^−1^ Er(NO_3_)_3_·5H_2_O was configured, and NH_4_F was dissolved in EG to form stock solution B with a concentration of 35.5 g L^−1^. Second, 1.5 g PAA, 0.17 g NaNO_3_, 0.38 g Bi(NO_3_)_3_·5H_2_O were dissolved 5 mL EG. Subsequently, 5 mL of stock solution A and 25 mL of stock solution B were added sequentially to the reaction system and vigorously stirred for 10 min. After stirring uniformly, the mixed solution was transfered to a Teflon-lined autoclave (50 mL) and set the reaction conditions of the oven to 150 °C for 9 h. After the reaction, the autoclave was naturally cooled and taken out. Next, the samples were washed four times with anhydrous ethanol, collected through centrifugation. Finally, the products were dried at room temperature. In addition, many comparative experiments were conducted to elucidate the formation mechanism of HNPs. The corresponding nanoparticles were obtained by adjusting the experimental parameters, including the amount of PAA (0–2 g), the amount of H_2_O (0–9 mL), and the reaction time (0–97 h).

### Characterization

Low-/high-resolution TEM images, elemental mapping images, SAED patterns, and EDS spectra were obtained using an FEI Tecnai G2S-Twin instrument with a field-emission gun operating at 200 kV. The crystal structures and phase purities of the nanoparticles were analyzed by powder XRD with a D8 Focus diffractometer (Bruker) with Cu Kα radiation (λ = 1.5418 Å) with an operation voltage and current maintained at 40 kV and 40 mA. XPS measurements were performed using a VG ESCALAB MKII spectrometer. FT-IR was measured on a PerkinElmer 580B IR spectrophotometer using the KBr pellet technique. The luminescence spectra at different temperatures were recorded using a fluorescence spectrofluorometer (Edinburgh Instruments, FLSP-920) equipped with a temperature controller and a 980 nm laser. The decay curve measurements were recorded and analyzed with a LeCroy WaveRunner 6100 1 GHz oscilloscope.

## Supplementary information

SUPPLEMENTAL MATERIAL
