# Supplementary material for: Hollow nanoparticles synthesized via Ostwald ripening and their upconversion luminescence-mediated Boltzmann thermometry over a wide temperature range
Source: Light Sci Appl. 2022 Jul 11;11:217. doi: 10.1038/s41377-022-00867-9 (PMC9273585; doi:10.1038/s41377-022-00867-9)
Supplement: Supplementary file 1 — SUPPLEMENTAL MATERIAL [file 41377_2022_867_MOESM1_ESM.docx]

**Supplementary Information for**

**Hollow nanoparticles synthesized via Ostwald ripening and their upconversion luminescence-mediated Boltzmann thermometry over a wide temperature range**

## Ran An^1^, Yuan Liang^1,2,3^, Ruiping Deng^1^, Pengpeng Lei^1,^*, Hongjie Zhang^1,2,4,^*

^1^State Key Laboratory of Rare Earth Resource Utilization, Changchun Institute of Applied Chemistry, Chinese Academy of Sciences, 5625 Renmin Street, Changchun 130022, China

^2^University of Science and Technology of China, Hefei 230026, China

^3^Ganjiang Innovation Academy, Chinese Academy of Sciences, Ganzhou, Jiangxi 341000, China

^4^Department of Chemistry, Tsinghua University, Beijing 100084, China

*Corresponding authors. Tel.: +86 431 85262127; fax: +86 431 85698041.

E-mail addresses: leipp@ciac.ac.cn (P. P. Lei), hongjie@ciac.ac.cn (H. J. Zhang).

**Fig. S1** The magnified X-ray diffraction (XRD) pattern of NaBiF_4_:Yb,Er (NBFYE) nanoparticles synthesized by adding 1.5 g polyacrylic acid (PAA) and 5 mL H_2_O to the reaction system for reaction 9 h.


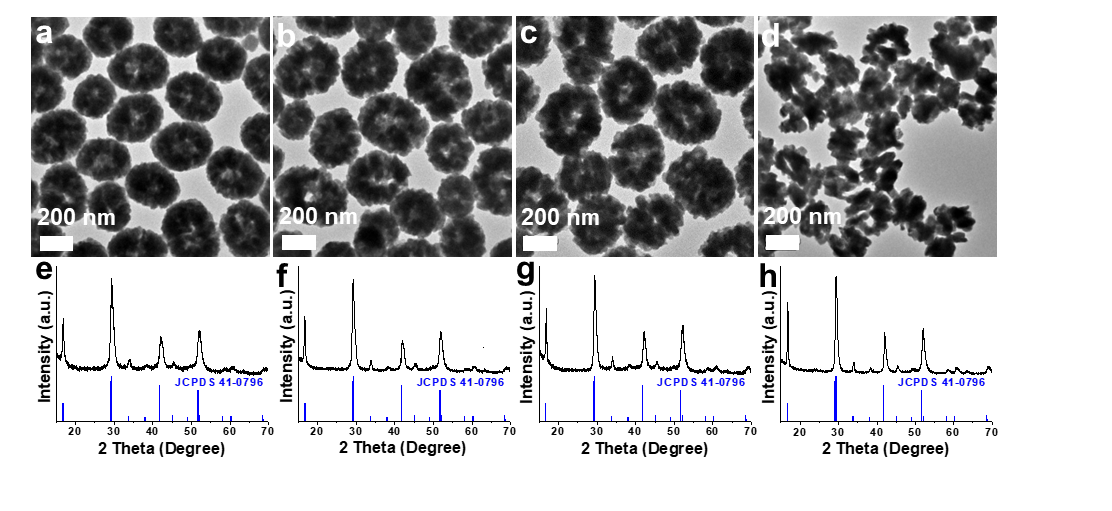


**Fig. S2** Transmission electron microscopy (TEM) images (a-d) and XRD patterns (e-h) of NBFYE nanoparticles synthesized by adding 1.5 g PAA and 5 mL H_2_O to the reaction system for different reaction times. (a, e) 13 h, (b, f) 21 h, (c, g) 29 h, and (d, h) 97 h.


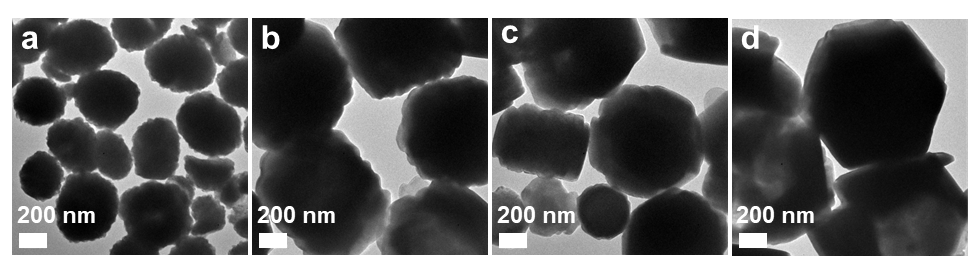


**Fig. S3** TEM images of NBFYE nanoparticles synthesized by adding different amounts of H_2_O to the reaction system without PAA. (a) 0 mL, (b) 1 mL, (c) 3 mL, and (d) 5 mL.


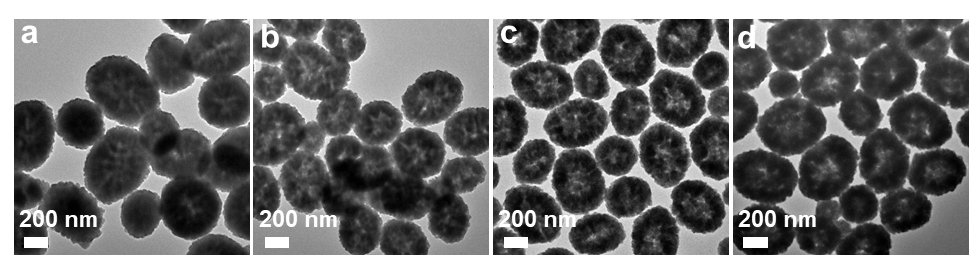


**Fig. S4** TEM images of NBFYE nanoparticles synthesized by adding different amounts of PAA to the reaction system in the presence of 5 mL H_2_O. (a) 0.5 g, (b) 1.0 g, (c) 1.5 g, and (d) 2.0 g.

**Fig. S5** Fourier transform infrared spectroscopy spectra of pure PAA and NBFYE hollow nanoparticles (HNPs) synthesized by adding 1.5 g PAA and 5 mL H_2_O to the reaction system.

**Fig. S6** XRD pattern of NBFYE nanoparticles synthesized by adding different amounts of H_2_O to the reaction system in the presence of 1.5 g PAA.


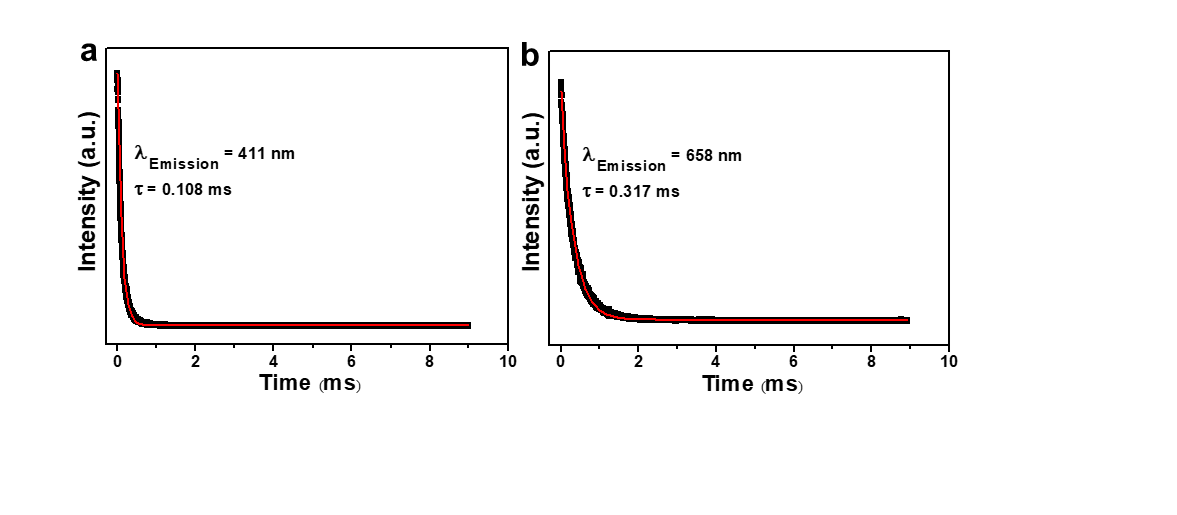


**Fig. S7** Time-resolved fluorescence decays of Er^3+^ ions emission at (a) 411 nm and (b) 658 nm of NBFYE HNPs synthesized by adding 1.5 g PAA and 5 mL H_2_O to the reaction system under 980 nm excitation.

**Fig. S8** UCL spectra of NaBiF_4_:Yb,Tm HNPs synthesized by adding 1.5 g PAA and 5 mL H_2_O to the reaction system under 980 nm laser excitation (power density: 4.85 W cm^-2^).

**Fig. S9** UCL spectra of NaBiF_4_:Yb,Ho HNPs synthesized by adding 1.5 g PAA and 5 mL H_2_O to the reaction system under 980 nm laser excitation (power density: 4.85 W cm^-2^).


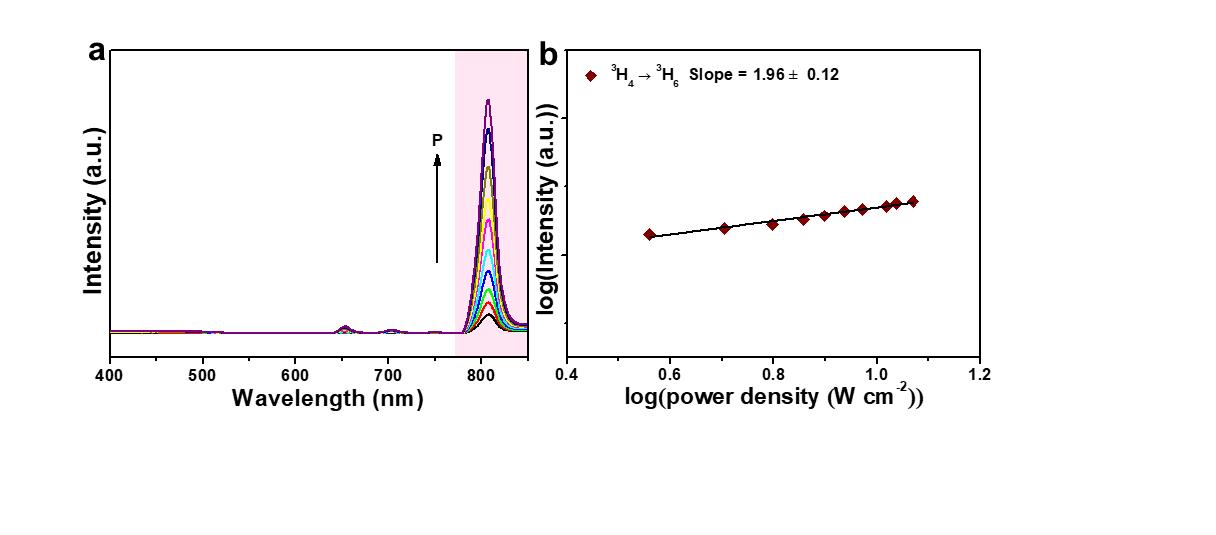


**Fig. S10** (a) UCL spectra of NaBiF_4_:Yb,Tm nanoparticles synthesized by adding 1.5 g PAA and 5 mL H_2_O to the reaction system under 980 nm laser excitation. The excitation power density ranges from 3.63 W cm^-2^ to 11.74 W cm^-2^. (b) Pump power dependence of UCL intensity of NaBiF_4_:Yb,Tm nanoparticles synthesized by adding 1.5 g PAA and 5 mL H_2_O to the reaction system under 980 nm laser excitation.


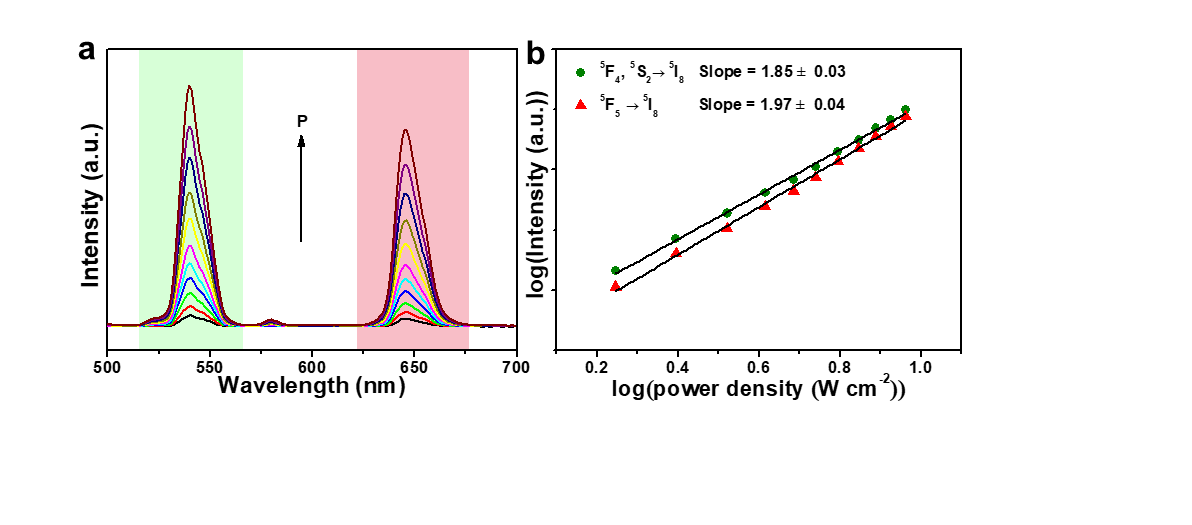


**Fig. S11** (a) UCL spectra of NaBiF_4_:Yb,Ho nanoparticles synthesized by adding 1.5 g PAA and 5 mL H_2_O to the reaction system under 980 nm laser excitation. The excitation power density ranges from 1.76 W cm^-2^ to 9.16 W cm^-2^. (b) Pump power dependence of UCL intensity of NaBiF_4_:Yb,Ho nanoparticles synthesized by adding 1.5 g PAA and 5 mL H_2_O to the reaction system under 980 nm laser excitation.

**Fig. S12** Time-resolved fluorescence decay of Tm^3+^ ions emission at 808 nm of NaBiF_4_:Yb,Tm HNPs synthesized by adding 1.5 g PAA and 5 mL H_2_O to the reaction system under 980 nm excitation.

The time-resolved fluorescence decay curve was fitted by a double exponential function: *I*(t)=*I*_0_+A_1_exp(-t/*τ*_1_)+A_2_exp(-t/*τ*_2_). The average decay times (*τ*) can be determined by the following formula: *τ* = (A_1_*τ*_1_^2^ + A_2_*τ*_2_^2^)/(A_1_*τ*_1_ + A_2_*τ*_2_).


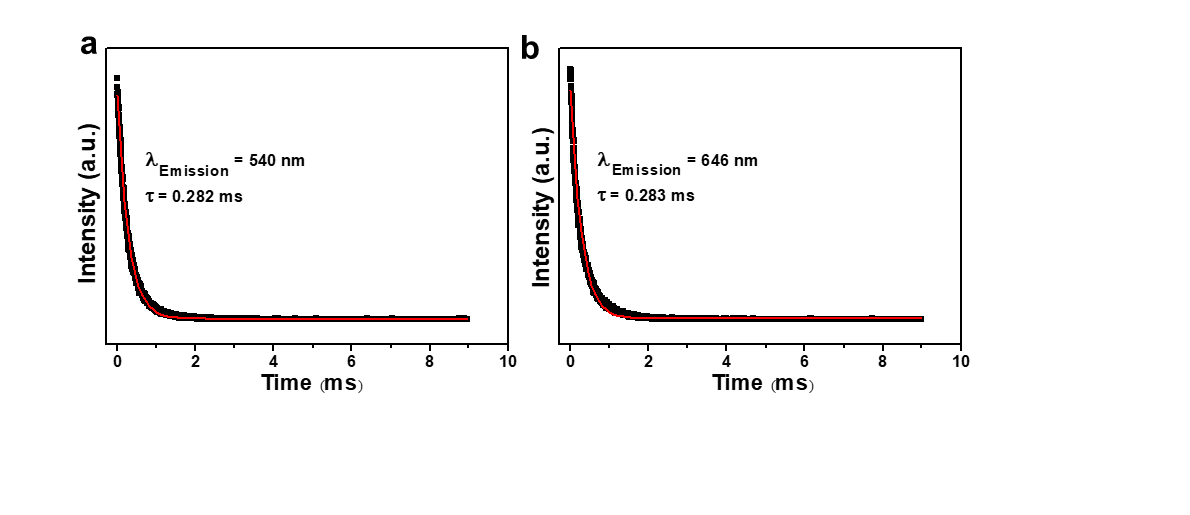


**Fig. S13** Time-resolved fluorescence decays of Ho^3+^ ions emission at 540 nm and 646 nm of NaBiF_4_:Yb,Ho HNPs synthesized by adding 1.5 g PAA and 5 mL H_2_O to the reaction system under 980 nm excitation.

The time-resolved fluorescence decay curves were fitted by a single exponential function: *I*(t)=*I*_0_+A_1_exp(-t/*τ*_1_). The average decay times (*τ*) is equal to *τ*_1_.

**Fig. S14** XRD pattern of NBFYE HNPs after temperature sensing performance test in the range of 223 to 548 K.
